# Prediction of Anal Cancer Recurrence After Chemoradiotherapy Using Quantitative Image Features Extracted From Serial ^18^F-FDG PET/CT

**DOI:** 10.3389/fonc.2019.00934

**Published:** 2019-09-27

**Authors:** Jiahui Wang, Hao Zhang, Michael Chuong, Kujtim Latifi, Shan Tan, Wookjin Choi, Sarah Hoffe, Ravi Shridhar, Wei Lu

**Affiliations:** ^1^Department of Radiation Oncology, University of Maryland Baltimore, Baltimore, MD, United States; ^2^Miami Cancer Institute, Baptist Hospital of Miami, Miami, FL, United States; ^3^Department of Radiation Oncology, Moffitt Cancer Center, Tampa, FL, United States; ^4^School of Automation, Huazhong University of Science and Technology, Wuhan, Hubei, China; ^5^Department of Medical Physics, Memorial Sloan Kettering Cancer Center, New York, NY, United States

**Keywords:** serial PET/CT, chemoradiation therapy, recurrence prediction, image analysis, anal cancer

## Abstract

We extracted image features from serial ^18^F-labeled fluorodeoxyglucose (FDG) positron emission tomography (PET) / computed tomography (CT) scans of anal cancer patients for the prediction of tumor recurrence after chemoradiation therapy (CRT). Seventeen patients (4 recurrent and 13 non-recurrent) underwent three PET/CT scans at baseline (Pre-CRT), in the middle of the treatment (Mid-CRT) and post-treatment (Post-CRT) were included. For each patient, Mid-CRT and Post-CRT scans were aligned to Pre-CRT scan. Comprehensive image features were extracted from CT and PET (SUV) images within manually delineated gross tumor volume, including geometry features, intensity features and texture features. The difference of feature values between two time points were also computed and analyzed. We employed univariate logistic regression model, multivariate model, and naïve Bayesian classifier to analyze the image features and identify useful tumor recurrent predictors. The area under the receiver operating characteristic (ROC) curve (AUC) was used to evaluate the accuracy of the prediction. In univariate analysis, six geometry, three intensity, and six texture features were identified as significant predictors of tumor recurrence. A geometry feature of Roundness between Post-CRT and Pre-CRT CTs was identified as the most important predictor with an AUC value of 1.00 by multivariate logistic regression model. The difference of Number of Pixels on Border (geometry feature) between Post-CRT and Pre-CRT SUVs and Elongation (geometry feature) of Post-CRT CT were identified as the most useful feature set (AUC = 1.00) by naïve Bayesian classifier. To investigate the early prediction ability, we used features only from Pre-CRT and Mid-CRT scans. Orientation (geometry feature) of Pre-CRT SUV, Mean (intensity feature) of Pre-CRT CT, and Mean of Long Run High Gray Level Emphasis (LRHGLE) (texture feature) of Pre-CRT CT were identified as the most important feature set (AUC = 1.00) by multivariate logistic regression model. Standard deviation (intensity feature) of Mid-CRT SUV and difference of Mean of LRHGLE (texture feature) between Mid-CRT and Pre-CRT SUVs were identified as the most important feature set (AUC = 0.86) by naïve Bayesian classifier. The experimental results demonstrated the potential of serial PET/CT scans in early prediction of anal tumor recurrence.

## Introduction

Anal cancer is a relatively uncommon malignancy. In the United States, the National Cancer Institute estimated 8,580 new cases and 1,160 deaths from anal cancer in 2018^1^. Chemoradiation therapy (CRT) is preferred over abdominoperineal resection for the treatment of anal cancer patients because of sphincter preservation, although surgery can be an effective salvage option (1–4). After CRT, early detection of tumor recurrence is important for initiating salvage surgery and preventing the spread of disease to distant sites (5, 6). Current guideline recommendations for treatment response evaluation after CRT includes serial digital rectal examination with biopsy of clinically progressive lesions, beginning 8–12 weeks after therapy is completed. However, early detection of residual and progressive disease can sometimes be challenging because of treatment-related mucositis and dermatitis that may limit adequate physical examination (5). Alternatively, as a non-invasive evaluation tool, anatomical imaging techniques (CT, ultrasound, and MRI) have been widely used in the tumor staging and treatment response evaluation. Because the region of anal tumors has similar intensity to the surrounding normal structures in the anatomical images and tumor margins may blend with surrounding normal tissues (5), these techniques may fail to accurately assess the presence of tumor.

Positron emission tomography (PET) scans provide metabolic information of tumors and can assist in differentiating recurrent tumors from surrounding tissue. As such, ^18^F-FDG PET scans obtained after CRT has been increasingly used for the anal cancer recurrence prediction (7, 8) alongside CT scans (^18^F-FDG PET/CT), which provide corresponding anatomic information. However, there are few reports in the literature about the usage of interim PET/CT scans (Mid-CRT) obtained during CRT to assess treatment response for anal cancer. In a recent paper of Hong et al. (9), they reported a anal cancer chemoradiation treatment evaluation study using pretreatment and interim PET/CT scans. Some commonly used standardized uptake value (SUV) based image features were evaluated, including maximum SUV (SUV_max_), mean SUV (SUV_mean_), metabolic tumor volume (MTV), and total lesion glycolysis (TLG). However, no correlation between tumor recurrence and relative change of those image features was detected.

Recent studies have shown that spatial PET/CT features are more informative than the commonly used SUV based measures (10, 11). In this study, instead of conventional SUV image features, comprehensive image features from both CT and SUV of serial PET/CT scans (Pre-CRT, Mid-CRT, and Post-CRT) were analyzed to identify useful image features for the prediction of anal cancer recurrence. In particular, we investigated the image features from Pre-CRT and Mid-CRT PET/CT scans only to explore their potential in the early prediction of tumor recurrence. The image features we used in this study include geometry, intensity, and texture features.

## Materials and Methods

### Patients and PET/CT Scans

IRB approval was obtained from both institutions involved in the analysis. The PET/CT scans used in this study were collected from Department of Radiation Oncology of one major cancer center in the United States for patients with non-metastatic squamous cell carcinoma of the anal canal treated with definitive CRT between 2008 and 2010. Seventeen patients were included in this study, including 4 recurrent and 13 non-recurrent. Clinical characteristics of the patients were shown in Table 1. One of the recurrent patients developed a locoregional recurrence and the other three were diagnosed with distant metastasis. The typical approach and clinical outcomes for managing anal cancer with CRT from this institution have been previously published (12). Patients received CRT for 32–50 total elapsed days (median 43 days) with initial prescription gross tumor volume (GTV) dose of 36–50 Gy and GTV boost dose of 0–22 Gy. The median GTV total dose was 56 Gy (range 50–62.5 Gy). Pre-CRT PET/CT imaging was performed 6–46 days before CRT (median 20 days), Mid-CRT PET/CT was performed 22–38 days (median 32 days) after the starting of CRT, and Post-CRT imaging was performed 42–141 days (median 88 days) after completion of CRT. All PET/CT scans were acquired with a Discovery-VCT (DVCT) scanner (GE Medical System, Milwaukee, USA). Each patient fasted for a minimum of 6 h before intravenous injection of 10 mCi ^18^F-FDG. Whole-body PET and CT imaging was started 90 min after tracer injection. The CT scans were acquired with 110 mAs and 120 kVp. Each slice had a matrix size of 512 × 512 pixels; the pixel was 1.37 × 1.37 mm with a 12-bit gray-level in Hounsfield Units (HU). The slice thickness was 3.27 mm. PET images were attenuation corrected with a matrix size of 128 × 128 pixels; the pixel was 5.47 × 5.47 mm. The slice thickness was 3.27 mm. To compare the PET-based image features between patients, standardized uptake value (SUV) was calculated on a voxel-by-voxel basis and a SUV image was created for each PET scan.

**Table 1:** Clinical characteristics of patients.

	**Non-recurrent patients**	**Recurrent patients**
*n*	13	4
**Gender**		
Male	5	1
Female	8	3
**Age**		
Median	53	49
Range	36 ~ 78	45 ~ 76
BMI	20.6 ~ 35.7	19.8 ~ 28.2
HIV+	2	0
Weight decrease during CRT (%)	−1.7 ~ 12.9	−1.9 ~ 22.4
**ECOG performance status**		
0	10	3
1	3	1
**T stage**		
1	3	0
2	6	1
3	4	1
4	0	2
**N stage**		
0	10	2
1	0	1
2	2	1
3	1	0
**AJCC stage (TNM stage)**		
2	3	0
3	7	1
4	0	2
5	3	1

An experienced radiation oncologist (MC) contoured the GTVs in the Pre-, Mid-, and Post-CRT PET/CT scans, respectively, using mainly CT while referring to PET. In general, anal tumors have similar intensity to the surrounding normal structures in CT scans. Thus, it would be hard for the radiation oncologists to contour the tumors accurately. FDG uptake of the tumors in PET scans could help in identify the tumor regions. However, high FDG uptake caused by non-tumor lesions, such as tissue inflammation, could affect the accuracy of delineated GTVs. In this study, we did not find such difficult cases.

### Image Registration

A rigid image registration followed by a B-Spline deformable image registration was used to align the Mid-CRT CT and Post-CRT CT to the Pre-CRT CT, respectively by maximizing their normalized cross correlation. To achieve higher registration accuracy in the tumor area, registration was constrained within a cuboid region, excluding irrelevant structures. The region ranged from the top of femoral head to the inferior pubic ramus, from the right lateral aspect of the right femoral head to the left lateral aspect of the left femoral head, and from the anterior border of the pubis to the most posterior border of sacrum.

The registration results were visually evaluated, and no obvious misalignments were observed. The resulting registration transform was applied to warp the manually delineated GTVs from Mid-CRT and Post-CRT scans to Pre-CRT scan, respectively. The Mid-CRT and Post-CRT SUV images were similarly warped to the Pre-CRT SUV image using the same transform as above, respectively. The following image analysis was performed in the same frame of reference, i.e., the Pre-CRT coordinate system.

### Extraction of Image Features

We used the Insight Segmentation and Registration Toolkit (ITK, National Library of Medicine; Bethesda, MD) to extract image features. Nineteen geometry features, nine intensity features, eight texture features based on co-occurrence matrix, and ten texture features based on run-length matrix were computed within the GTVs in the Pre-, Mid-, and Post-CRT CT and SUV images, respectively. The difference or change of feature values between two time points were also computed, including Diff1 = Mid-CRT - Pre-CRT, Diff2 = Post-CRT - Mid-CRT, and Diff3 = Post-CRT - Pre-CRT. The detailed definition of these features was described in Appendix A.

#### Geometry Features

Geometry features described the shape, size, or relative position of a tumor. Nineteen geometry features were computed (13, 14), including volume, major axis length, minor axis length, eccentricity, elongation, orientation, bounding box volume, oriented bounding box volume, equivalent spherical perimeter, equivalent spherical radius, ferret diameter, number of lines, number of pixel on border, perimeter, perimeter on border, perimeter on border ratio, physical size, region elongation, and roundness. For instance, Roundness (*R*) is defined by

$$\begin{array}{l} \text{R}=A/v \end{array}$$

where *v* is the surface area of the GTV, *A* is the surface area of the hyper-sphere with the same volume of the GTV.

#### Intensity Features

Nine intensity features were computed based on the intensity (CT number in CT images and SUV in PET images) of all voxels within the GTV, including minimum, maximum, mean, standard deviation, sum, median, skewness, kurtosis, and variance.

#### Texture Features

Texture features quantify the spatial patterns of tumor from images (15). In each CT or SUV image, the intensity was first normalized into 64 gray levels. The texture features were computed based on the gray level co-occurrence matrix (GLCM) (16–18) and gray level run-length matrix (GLRM) (18, 19).

An element of a GLCM measures the number of two specified gray levels separated by a given distance in a specified direction (16–18). After the construction of the GLCM, the following eight frequently used features were computed (16–18): Energy, entropy, correlation, inverse difference moment, inertia, cluster shade, cluster prominence, Haralick correlation. Each GLCM feature was computed in 13 directions (in 3D) with a distance of one voxel between the pair of voxels. The feature was then averaged over the 13 directions. The standard deviation of each feature over the 13 directions was computed as well.

An element of a GLRM measures the number of “runs” with a specific length and specific gray level (18, 19), where a “run” is defined as a block of consecutive voxels with the same gray level in a specific direction. In this study, we set the maximum allowed length of runs to the length of the minimum bounding box of the GTV. After the construction of the GLRM, the following ten frequently used features were computed: short run emphasis (SRE), long run emphasis (LRE), gray level non-uniformity (GLN), run length non-uniformity (RLN), low gray level run emphasis (LGLRE), high gray level run emphasis (HGLRE), short run low gray level emphasis (SRLGLE), short run high gray level emphasis (SRHGLE), long run low gray level emphasis (LRLGLE), long run high gray level emphasis (LRHGLE). Each GLRM feature was also computed in 13 directions. The feature was then averaged over the 13 directions. The standard deviation of each feature over the 13 directions was computed as well.

### Predictive Model Construction

For this study the binary response variable of interest is the recurrence of anal cancer, coded 1 = recurrence and 0 = non-recurrence. The area under the receiver operating characteristic (ROC) curve (AUC) was used to evaluate the accuracy of the prediction.

Firstly, we used a univariate logistic regression model (20) to identify individually significant image features for predicting recurrence. The AUC of each image feature was obtained and *p*-value was calculated with Wilcoxon rank-sum test (21). A cut-off *p*-value of 0.05 was used to identify significant tumor recurrence predictors, i.e., an image feature was identified as a significant predictor, if its *p*-value was lower than 0.05.

Secondly, we used a multivariate logistic regression model (20) to select the most significant feature set (as single feature or multiple features) for predicting recurrence. The initial null hypothesis was that there was no relationship between the image features and recurrence. When multivariate logistic regression was used, null hypotheses of adding image features to the multiple logistic regression do not improve the prediction accuracy any more than expected by chance were tested. Again, *p*-value of 0.05 from the null hypotheses was used to select the most significant feature set.

Lastly, we used an advanced pattern classification framework of naïve Bayesian classifier (22) for predicting recurrence using the identified feature set as input. Due to the small patient cohort, leave-one-out cross-validation was used. Let *C* be the outcome (recurrence) class, which is modeled as a random variable, and let *X* be a vector of random variables denoting the input features. Further, let *c* and *x* represent particular class of *C* and particular observed value of *X*. Our model uses Bayes' rule to compute the probability of each class given the observed values as,

(1) $$\begin{array}{l} p\left(C=c|X=x\right)=\frac{p\left(C\;=\;c\right)p\left(X\;=\;x|C\;=\;c\right)}{p\left(X\;=\;x\right)} \end{array}$$

Because in naïve Bayesian classifier the features are assumed to be conditionally independent, we have

$$\begin{array}{l} p\left(X=x|C=c\right)=\underset{i}{\prod }p\left(X_{i}=x_{i}|C=c\right) \end{array}$$

which is simple to estimate from training data as well as to compute for test data. For example, for each recurrent class and continuous image feature, we will estimate the mean and standard deviation of the feature given the class. Traditionally a single Gaussian distribution assumption was used when estimating the mean and standard deviation. Here this assumption was eliminated in favor of kernel density estimation, but still maintaining the independence assumption (22). Finally, the probability computed from (1) is used to determine the most probable class.

## Results

### Univariate Analysis of Logistic Regression Model

Six geometry features, three intensity features, and six texture features (three co-occurrence matrix features and three run-length matrix features) were identified as individually significant predictors (*p* ≤ 0.05) to differentiate recurrence and non-recurrence using the univariate logistic regression model. The identified predictors were listed in Table 2.

**Table 2:** Selected anal cancer recurrence predictors from all the image features by univariate logistic regression model.

**Features**	**^a^Association**	**AUC**	***p*-value**
Diff3 Roundness	+	1.00	0.00
Post-CRT Roundness	–	0.96	0.00
Diff2 Roundness	+	0.90	0.01
Diff3 Perimeter on Border Ratio	–	0.77	0.02
Diff3 CT Minimum	+	0.85	0.02
Post-CRT CT SD of Correlation	–	0.77	0.03
Post-CRT Major Axis Length	–	0.81	0.03
Diff3 CT Mean of Inverse Difference Moment	–	0.83	0.03
Post-CRT CT Elongation	–	0.83	0.04
Diff3 CT Mean of Short Run Emphasis	+	0.83	0.04
Post-CRT CT Minimum	+	0.62	0.04
Post-CRT CT Mean of Inverse Difference Moment	–	0.63	0.05
Post-CRT SUV SD of Cluster Shade	+	0.69	0.05
Diff3 CT Mean	+	0.79	0.05
Diff1 CT SD of Long Run High Gray Level Emphasis	–	0.83	0.05

a *Association = “+” indicates the larger a feature, the more likely tumor recurrent; Association = “−” indicates the larger a feature, the less likely tumor recurrent*.

### Multivariate Analysis of Logistic Regression

By applying the multivariate logistic regression model, the Diff3 of roundness of CT, i.e., the difference in tumor roundness between Post-CRT and Pre-CRT, was identified as the most powerful predictor with an AUC of 1.0. Using Pre-CRT and Mid-CRT scans only, Orientation of Pre-CRT SUV and LRHGLE of Pre-CRT CT were selected as the most important feature set with a high AUC of 1.00. The results were shown in Table 3.

**Table 3:** Selected anal cancer recurrence predictors (correlation to the recurrence in parentheses) by multivariate logistic regression model.

	**Using Pre-CRT, Mid-CRT, and Post-CRT**	**Using Pre-CRT and Mid-CRT**
Features	Diff3 CT Roundness (0.83)	Pre-CRT SUV Orientation (−0.31), Pre-CRT CT Mean (−0.15), Pre-CRT CT Mean of Long Run High Gray Level Emphasis (0.41)
AUC	1.00	1.00

### Naïve Bayesian Classifier Based Analysis

By applying the naïve Bayesian classifier, Diff3 of Number of Pixels on Border of SUV and Elongation of Post-CRT CT were identified as the most useful feature set with AUC = 1.00. Using Pre-CRT and Mid-CRT scans only, standard deviation of Mid-CRT SUV, Diff1 of Mean of LRHGLE of SUV were selected as the most important feature set with an AUC of 0.86. The results were shown in Table 4.

**Table 4:** Selected anal cancer recurrence predictors (correlation to the recurrence in parentheses) by naïve Bayesian classifier.

	**Using Pre-CRT, Mid-CRT, and Post-CRT**	**Using Pre-CRT and Mid-CRT**
Features	Diff3 SUV Number of Pixels on Border (−0.07) and Post-CRT CT Elongation (−0.28)	Mid-CRT SUV Standard Deviation (−0.15), Diff1 SUV Mean of Long Run High Gray Level Emphasis (0.16)
AUC	1.00	0.86

## Discussion

Almost all the predictors identified by univariate logistic regression model, multivariate logistic regression model, and naïve Bayesian classifier were derived from Post-CRT scans or from Diff3 (Post-CRT - Pre-CRT). To investigate the early prediction ability of the features, we applied the multivariate logistic regression model and naïve Bayesian classifier by using features from Pre-CRT and Mid-CRT scans and Diff1 only. As shown in Table 3, Orientation of Pre-CRT SUV, Mean of Pre-CRT CT, and Mean of LRHGLE of Pre-CRT CT were identified as the most useful feature set by the multivariate logistic regression model with an AUC of 1.0. As shown in Table 4, standard deviation of Mid-CRT SUV and Diff1 of Mean of LRHGLE of SUV were identified as the most useful feature set by the naïve Bayesian classifier with an AUC of 0.86. These results demonstrated the potential of Pre-CRT and Mid-CRT PET/CT scans for the early predication of anal cancer recurrence.

**Figure 1:**
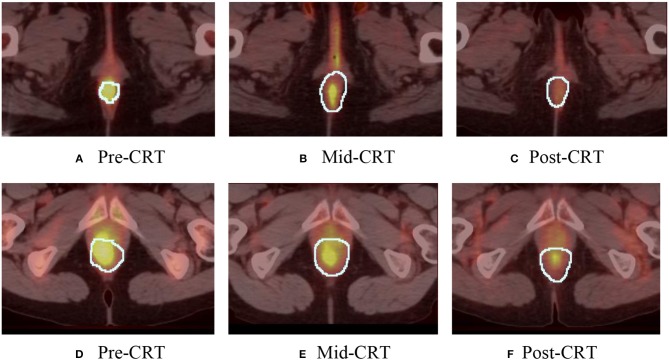
Manually delineated tumor contour (white) in Pre-CRT, Mid-CRT, and Post-CRT scans of a non-recurrent patient **(A–C)** and a recurrent patient **(D–F)** Post-CRT. Roundness showed different changing patterns (in Diff3 = Post-CRT – Pre-CRT) between recurrent and non-recurrent groups.

One geometry feature Diff3 of roundness, has been identified as the most useful predictor by both univariate and multivariate logistic regression models. Roundness measures how similar the shape of a tumor is to a sphere with range [0, 1]. A larger value of roundness means higher similarity to a sphere. As shown in Figure 1 and Table 5 the roundness of all four recurrent tumors increased from Pre-CRT to Post-CRT by 0.05 or more, with a mean increase of 0.08, whereas the roundness of the 13 non-recurrent tumors either decreased (11 tumors), or did not change (2 tumors), or increased slightly by 0.01 (1 tumor), with a mean decrease of 0.06. Therefore, by using Diff3 of roundness only, we were able to correctly predict all cases with an AUC of 1.0.

**Table 5:** The roundness of each patient at Pre-CRT, Mid-CRT, and Post-CRT and their differences.

**Recurrent Status (1 = recurrent, 0 = non-recurrent)**	**Pre**	**Mid**	**Post**	**Diff1**	**Diff2**	**Diff3**
0	0.78	0.70	0.67	−0.08	−0.04	−0.12
0	0.68	0.64	0.69	−0.04	0.05	0.01
0	0.80	0.61	0.75	−0.19	0.14	−0.05
0	0.72	0.73	0.70	0.01	−0.04	−0.03
0	0.80	0.64	0.70	−0.15	0.05	−0.10
0	0.72	0.70	0.62	−0.01	−0.08	−0.09
0	0.70	0.78	0.58	0.08	−0.20	−0.12
0	0.84	0.67	0.76	−0.17	0.09	−0.09
0	0.78	0.73	0.78	−0.04	0.04	0.00
0	0.73	0.80	0.70	0.06	−0.10	−0.03
0	0.88	0.76	0.77	−0.12	0.01	−0.11
0	0.75	0.72	0.74	−0.02	0.02	0.00
0	0.76	NA	0.72	NA	NA	−0.04
1	0.68	0.66	0.76	−0.02	0.10	0.08
1	0.78	0.69	0.83	−0.09	0.14	0.05
1	0.67	0.71	0.78	0.04	0.07	0.11
1	0.72	0.73	0.80	0.01	0.07	0.08
Mean of non-recurrent	0.76	0.71	0.71	−0.06	0.00	−0.06
Mean of recurrent	0.71	0.70	0.79	−0.01	0.10	0.08

*Diff1 = Mid-CRT – Pre-CRT; Diff2 = Post-CRT – Mid-CRT; Diff3 = Post-CRT – Pre-CRT*.

**Figure 2:**
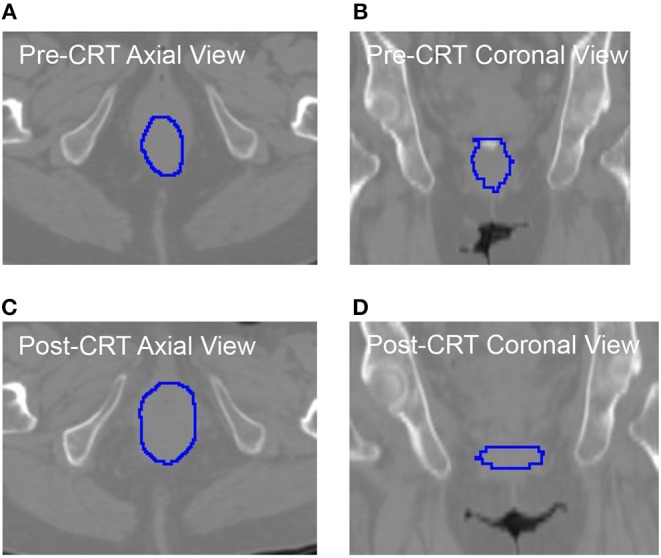
The CT images with manually delieated tumor contour of anal cancer patient with no tumor recurrence. The tumor regressed in coronal directions **(B)** vs. **(D)**. However, it progressed in axial direction **(A)** vs. **(C)**. The roundness of this tumor was changed from 0.70 (Pre-CRT) to 0.58 (Post-CRT).

As shown in Table 5, the roundness of many of the non-recurrent tumors decreased from Pre-CRT to Post-CRT and the roundness of recurrent tumors increased from Pre-CRT to Post-CRT. The difference in roundness may reflect that the normal anal canal has a low roundness value, which would be consistent with decreased anal tumor burden; to the contrary, higher gross tumor burden would have a higher roundness value. Figure 2 shows an example of the comparison between Pre-CRT and Post-CRT of a non-recurrent tumor. Its roundness decreased from 0.70 to 0.58. The tumor regressed significantly in coronal direction. However, it enlarged in axial direction on CT. We further investigated all the tumors in our dataset. For non-recurrent patients, the changes of tumor size were mainly in superior-inferior direction. However, the changes were not consistent, i.e., some tumors decreased, and other tumors increased in superior-inferior direction. On the other hand, the changes in axial plane were generally quite small. For recurrent patients, changes could be observed in both axial plane and superior-inferior direction. However, we did not find a consistent pattern in the trend of size changes either. It is important to note that the radiation oncologist who delineated the tumor volumes was blinded to the prior contours while contouring the follow up scans, which could have affected the consistency of the volumes over time including perceived enlargement of delineated tumor regions in follow up scans of non-recurrent patients. Therefore, the recurrence prediction purely based on the geometry measurements, such as roundness, volume, etc., may not be reliable enough. Intensity and texture-based imaging features would be useful complementary to the geometry measurements in the recurrence prediction. In addition, this experimental result could raise a hypothesis—tumors may regress in a non-uniform manner after CRT. The tumors with positive response to the treatment may have regressed asymmetrically, which also may have contributed to their roundness measurement decreasing. We were unable to find other published literature about directional tumor regression after CRT and therefore warrant further evaluation.

In addition to roundness, some other features were identified as recurrence predictors by multivariate logistic regression model (Table 3) and by naïve Bayesian classifier (Table 4). Each of these features had low correlation (<0.50) to tumor recurrence and was weak classifier by itself. However, the performance can be improved significantly by systematically combining a number of weak classifiers (23) and using well-designed training procedure, such as the multivariable logistic regression model and the naïve Bayesian classifier in this study. By use of these combined features, relatively high performance in the tumor recurrence prediction was achieved (high AUC values) as shown in Tables 3, 4.

Traditional naïve Bayesian classifier assumes that numeric features are generated by a single Gaussian distribution. It is a reasonable approximation to many real-world applications, but not always the best. Our approach extended the traditional naïve Bayesian by eliminating the single Gaussian assumption, which certainly could be violated in cancer recurrence prediction. Another advantage of the method is that it does not suffer from the high dimensionality of the model. This is because features are assumed to be conditionally independent in Naïve Bayesian classifier, so that the curse-of-dimensionality can be avoided by allowing the join distribution to be decomposed. Naïve Bayesian classifier is also closely related to the widely used logistic regression classifier. While naïve Bayesian classifier fits a probability that optimizes the joint likelihood, logistic regression fits the same probability model that optimizes the conditional probability. It has been shown that in some practical cases naïve Bayesian can outperform logistic regression because it converges faster (24).

One limitation of this study was that this was a retrospective analysis of a small patient cohort (*n* = 17), particularly only 4 patients with recurrence. This was a small, unbalanced dataset for reliable prediction. Although we used cross-validations to avoid potential over fitting, the predictive accuracy and stability of the model should be validated in a larger and independent patient cohort. Another limitation is the lack of standard dose prescription for each patient. The total dose a patient received was based subjectively on tumor response as per Mid-CRT PET. Finally, it was hard to provide biological or clinic explanations for why the extracted image features were important for recurrence prediction.

## Conclusions

Early prediction of tumor persistence or recurrence using PET/CT scans obtained prior to or during CRT for anal cancer may be possible through analysis of quantitative imaging features. Additional study is warranted in a larger patient population to confirm our findings. A future study to investigate the correlation between clinical characteristics (e.g., T staging, N staging, radiation dose, etc.) and the image features is needed as well.

## Data Availability Statement

The datasets generated for this study are available on request to the corresponding author.

## Ethics Statement

The studies involving human participants were reviewed and approved by Department of Radiation Oncology, University of Maryland Baltimore and Department of Radiation Oncology, Moffitt Cancer Center. Written informed consent for participation was not required for this study in accordance with the national legislation and the institutional requirements.

## Author Contributions

WL, MC, and JW contributed conception and design of the study. MC, KL, SH, and RS collected experimental data, evaluated the tumor recurrence status, and created clinical indices of the dataset. MC delineated gross tumor volume for each patient. JW, WC, and ST contributed to the computation of image features. HZ and WC performed statistical analysis. JW, HZ, MC, WC, and WL wrote the draft of the manuscript. All authors contributed to manuscript revision, read, and approved the submitted version.

### Conflict of Interest

The authors declare that the research was conducted in the absence of any commercial or financial relationships that could be construed as a potential conflict of interest.

## Footnotes

^1^http://seer.cancer.gov/statfacts/html/anus.html
