# Supplementary material for: Prediction of Anal Cancer Recurrence After Chemoradiotherapy Using Quantitative Image Features Extracted From Serial 18F-FDG PET/CT
Source: Front Oncol. 2019 Sep 27;9:934. doi: 10.3389/fonc.2019.00934 (PMC6777412; doi:10.3389/fonc.2019.00934)
Supplement: Supplementary file 1 [file Data_Sheet_1.docx]

# Appendix A. Image Features

**A.1. Geometry features**

The geometry features used in this study were computed by Insight Segmentation and Registration Toolkit (ITK, <https://itk.org>) libraries. The detailed definition and computation of the features can be found in [[13](#_ENREF_13), [14](#_ENREF_14)]. The geometry features identified as significant predictors by logistic regression model or naïve Bayesian classifier (Tables 2 - 4) were listed below

- Major axis length: the length of the major axis of hyper-ellipsoid. The fitting algorithm of hyper-ellipsoid could be found in [[12](#_ENREF_12)].
- Elongation: the ratio of the major axis length by the minor axis length of the hyper-ellipsoid.
- Number of pixels on border: the number of voxels on the surface of the GTV
- Perimeter on border ratio: the ratio of the surface area of the bounding box of GTV and the real surface area of GTV.
- Orientation: the angle between major axis and the origin. Please refer to [[13](#_ENREF_13), [14](#_ENREF_14)] for the definition of major axis and the detail of the computation.
- Roundness = $A/v$

where *v* is the volume of the GTV, *A* is the hyper-sphere with the same volume of the GTV

**A.2. Intensity features**

The definition of nine intensity features were listed below

- Minimum: minimum voxel value for the GTV
- Maximum: maximum voxel value for the GTV
- Mean: average voxel value of the GTV
- Standard deviation: the standard deviation of the voxel values in the GTV
- Sum: the sum of all the voxel values in the GTV
- Median: median of the voxel values in the GTV
- Skewness: $\frac{1}{s^{3}}\sum_{i=1}^{n} {\left( x_{i}-\mu\right)^{3}}/N$

where *n* the total number of voxels in the GTV, *x_i_* is the voxel value of a voxel, *μ* is the mean of voxel value of the GTV, and *s* is the standard deviation of the voxel values in the GTV.

- Kurtosis: $\frac{1}{s^{4}}\sum_{i=1}^{n} {\left( x_{i}-\mu\right)^{4}}/N-3$

where *n* the total number of voxels in the GTV, *x_i_* is the voxel value of a voxel, *μ* is the mean of voxel value of the GTV, and *s* is the standard deviation of the voxel values in the GTV.

- Variance: $\frac{1}{n}\sum_{i=1}^{n} \left( x_{i}-\mu\right)^{2}$

where *n* the total number of voxels in the GTV, *x_i_* is the voxel value of a voxel, and *μ* is the mean of voxel value of the GTV

**A.3. Co-occurrence matrix texture features**

The definition of eight co-occurrence matrix texture features [[16](#_ENREF_16)] were listed below. Each element e(i, j) of the co-occurrence matrix was first transformed to normalized probability p(i., j).

$$P\left( i,j \right)=\frac{e\left( i,j \right)}{\sum_{i=1}^{N} \sum_{j=1}^{N} e\left( i,j \right)}$$

where *N* is the dimension of the co-occurrence matrix.

- Entropy = $-\sum_{i=1}^{N} \sum_{j=1}^{N} P\left( i,j \right)lgP\left( i,j \right)$
- Energy =$\sqrt{\sum_{i=1}^{N} \sum_{j=1}^{N} {P\left( i,j \right)}^{2}}$
- Correlation = $\sum_{i=1}^{N} \sum_{j=1}^{N} \frac{\left( i-\mu_{x})(j-\mu_{y} \right)P\left( i,j \right)}{\sigma_{x}\sigma_{y}}$

where $\mu_{x}=\sum_{i=1}^{N} i\sum_{j=1}^{N} P\left( i,j \right)$, $\mu_{y}=\sum_{i=1}^{N} \sum_{j=1}^{N} jP\left( i,j \right)$, $\sigma_{x}=\sqrt{\sum_{i=1}^{N} \left( i-\mu_{x} \right)^{2}\sum_{j=1}^{N} P\left( i,j \right)}$, $\sigma_{y}=\sqrt{\sum_{j=1}^{N} \left( j-\mu_{y} \right)^{2}\sum_{i=1}^{N} P\left( i,j \right)}$

- Inverse difference moment = $\sum_{i=1}^{N} \sum_{j=1}^{N} \frac{1}{1+\left( i-j \right)^{2}}P\left( i,j \right)$
- Inertia = $\sum_{i=1}^{N} \sum_{j=1}^{N} P\left( i,j \right)\left( i-j \right)^{2}$
- Cluster shade = $\sum_{i=1}^{N} \sum_{j=1}^{N} P\left( i,j \right)\left( i+j-\mu_{x}-\mu_{y} \right)^{3}$
- Cluster prominence = $\sum_{i=1}^{N} \sum_{j=1}^{N} P\left( i,j \right)\left( i+j-\mu_{x}-\mu_{y} \right)^{4}$
- Haralick correlation = $\sum_{i=1}^{N} \sum_{j=1}^{N} \frac{\left( ij \right)P\left( i,j \right)-\mu_{x}\mu_{y}}{\sigma_{x}\sigma_{y}}$

**A.4. Run length matrix texture features**

The definition of ten run length matrix texture features [[19](#_ENREF_19)] were listed below

- Short run emphasis (SRE) = ${\sum_{i=1}^{N_{g}} \sum_{j=1}^{N_{r}} {p\left( i,j \right)}/{j^{2}}}/n$

where *p(i,j)* is the *(i,j)* th entry of the run length matrix, *N_g_* is the number of the gray level of the gray levels in the image, *N_r_* is the number of different run lengths that occurred in the image, and $n = \sum_{i=1}^{N_{g}} \sum_{j=1}^{N_{r}} p\left( i,j \right)$ .

- Long run emphasis (LRE) = ${\sum_{i=1}^{N_{g}} \sum_{j=1}^{N_{r}} j^{2}p\left( i,j \right)}/n$
- Gray level non-uniformity (GLN) = ${\sum_{i=1}^{N_{g}} {(\sum_{j=1}^{N_{r}} p\left( i,j \right)}^{2})}/n$
- Run length non-uniformity (RLN) = ${\sum_{j=1}^{N_{g}} {(\sum_{i=1}^{N_{r}} p\left( i,j \right)}^{2})}/n$
- Low gray level run emphasis (LGLRE) = ${\sum_{i=1}^{N_{g}} \sum_{j=1}^{N_{r}} {p\left( i,j \right)}/{i^{2}}}/n$
- High gray level run emphasis (HGLRE) = ${\sum_{i=1}^{N_{g}} \sum_{j=1}^{N_{r}} i^{2}p\left( i,j \right)}/n$
- Short run low gray level emphasis (SRLGLE) = ${\sum_{i=1}^{N_{g}} \sum_{j=1}^{N_{r}} {p\left( i,j \right)}/{(i^{2}{\cdot j}^{2}})}/n$
- Short run high gray level emphasis (SRHGLE) = ${\sum_{i=1}^{N_{g}} \sum_{j=1}^{N_{r}} {p\left( i,j \right){\cdot i}^{2}}/{j^{2}}}/n$
- Long run low gray level emphasis (LRLGLE) = ${\sum_{i=1}^{N_{g}} \sum_{j=1}^{N_{r}} {p\left( i,j \right){\cdot j}^{2}}/{i^{2}}}/n$
- Long run high gray level emphasis (LRHGLE) = ${\sum_{i=1}^{N_{g}} \sum_{j=1}^{N_{r}} p\left( i,j \right){\cdot i}^{2}{\cdot j}^{2}}/n$
